# A Human Skin Explant Test as a Novel In Vitro Assay for the Detection of Skin Sensitization to Aggregated Monoclonal Antibodies

**DOI:** 10.3390/toxics12050332

**Published:** 2024-04-30

**Authors:** Ana Martins-Ribeiro, Arathi Kizhedath, Shaheda Sameena Ahmed, Jarka Glassey, Abbas Ishaq, Matthew Freer, Anne Mary Dickinson

**Affiliations:** 1 Alcyomics Ltd., The Biosphere, Draymans Way, Newcastle Helix, Newcastle Upon Tyne NE4 5BX, UK; anappmribeiro@gmail.com (A.M.-R.); matthew.freer@alcyomics.com (M.F.); 2 Translational and Clinical Research Institute Faculty of Medical Sciences, Newcastle University, Newcastle Upon Tyne NE2 4HH, UK; 3 Chemical Engineering and Advanced Materials, Newcastle University, Newcastle Upon Tyne NE1 7RU, UK

**Keywords:** mAb aggregation, skin sensitization, immunotoxicity, in vitro test, T-cell proliferation, cell death, skin explant, safety assessment, immunotoxicity

## Abstract

**Introduction:** Monoclonal antibodies (mAbs) are important therapeutics. However, the enhanced potential for aggregation has become a critical quality parameter during the production of mAbs. Furthermore, mAb aggregation may also present a potential health risk in a clinical setting during the administration of mAb therapeutics to patients. While the extent of immunotoxicity in patient populations is uncertain, reports show it can lead to immune responses via cell activation and cytokine release. In this study, an autologous in vitro skin test designed to predict adverse immune events, including skin sensitization, was used as a novel assay for the assessment of immunotoxicity caused by mAb aggregation. **Material and Methods**: Aggregation of mAbs was induced by a heat stress protocol, followed by characterization of protein content by analytical ultra-centrifugation and transmission electron microscopy, revealing a 4% aggregation level of total protein content. Immunotoxicity and potential skin sensitization caused by the aggregates, were then tested in a skin explant assay. **Results:** Aggregated Herceptin and Rituximab caused skin sensitization, as shown by histopathological damage (grade II–III positive response) together with positive staining for Heat Shock Protein 70 (HSP70). Changes in T cell proliferation were not observed. Cytokine analysis revealed a significant increase of IL-10 for the most extreme condition of aggregation (65 °C at pH3) and a trend for an overall increase of IFN-γ, especially in response to Rituximab. **Conclusions:** The skin explant assay demonstrated that aggregated mAbs showed adverse immune reactions, as demonstrated as skin sensitization, with histopathological grades II-III. The assay may, therefore, be a novel tool for assessing immunotoxicity and skin sensitization caused by mAb aggregation.

## 1. Introduction

mAb industrial production is a complex process where several stress factors must be taken into consideration during industrial scale-up and manufacturing of therapeutic antibody proteins. Temperature is one such critical parameter since thermal changes can affect the conformational structure and stability of the IgG mAb molecule [1]. This poses a serious cost issue due to production failure, as it can lead to epitope loss and loss of antibody efficacy together with irreversible aggregation events. At high temperatures, the disulphide bridges, binding the polypeptide chains of the antibody, start to weaken and denature. This leads to an unfolding of the overall structure and an unstable conformational state [1,2,3,4]. pH can also induce antibody aggregation via protein instability [2,5,6]. Acid-induced changes can promote loss of native structure by means of secondary structure refolding [6]. More specifically, one study showed [2] that while the Fab fragment was very sensitive to heat stress, the Fc region was more sensitive to acidic pH. Physical or mechanical stress can also potentiate antibody aggregation. This stress can be caused by agitation during manufacture [7], storage [8], shipping, or shaking during administration of the antibody [9]. Commercially available mAbs are formulated to be very stable endpoint products, but exposure to temperature, pH, or stirring stress factors can compromise mAb stability, ultimately potentially having effects on clinical safety.

Considering the size of protein aggregates (ranging from 100 to 1000 kDa), their characterization becomes quite complex. Therefore, multi-technique analysis or a combination approach provides more reliable information. New analytical techniques, include, analytical ultracentrifugation (AUC) [10], size exclusion chromatography-multi angle light scattering (SEC-MALS) [11], and asymmetric flow field-flow fractionation multi-angle light scattering [12].

Asymmetric flow field-flow fractionation (AF4) is a technique that offers the possibility to physically separate biomolecules across a wide size range. AF4 can be used in combination with different types of detectors depending on the information required and becomes especially powerful when combined with multi-angle light scattering (MALS) [13] and (AF4-MALS) [14] are the current most reliable methods for quantifying low levels of protein aggregates. AUC allows for the quantification of protein aggregation and the formulation of large supramolecular complexes based on their sedimentation properties [15]. Sedimentation velocity (SV)-AUC is a hydrodynamic approach that provides details of particle mass and shape and is particularly useful for studying multicomponent irreversible and reversible mixtures of species [15]. In this study, SV-AUC was used to explore the formation of stable and metastable oligomers, irreversible aggregates, and degradation products due to storage and thermal stress.

There is a concern that protein and antibody aggregation may represent a potentially serious cause of adverse immunotoxicity in humans when administered therapeutically. For example, one study investigating the instability of antibodies during aerosolization of lung mucosa in mice found the development of aggregates, which led to pro-inflammatory and cytotoxic effects [16]. The Clinical Oncology Society of Australia Position Statement [17] also reviews the safe handling of monoclonal antibodies in the health care setting where distinct exposure mechanisms, dermal, mucosal, inhalation, and oral, were discussed with regard to health risk, e.g., dermatitis and skin sensitization.

Indeed, the aggregation of proteins is a complex multifactorial process that includes conformational change, nucleation, and the growth of protein particles. Protein-based vaccines, for example, are a demonstration of when immunogenicity is desirable as they are more effective at eliciting an immune response as aggregated in an adjuvant particle. However, in the case of immunogenicity, the aggregation of proteins can be undesirable and cause an adverse immune response [18]. In this instance, immunotoxicity can be defined as an adverse immune activation event, including T cell proliferation and cytokine stimulation caused by aggregated mAb therapeutics. However, little is known about mAb aggregation following patient administration. Indeed, previous studies have shown that there is a significant chance that mAb preparations alone will cause some degree of immunotoxicity [19,20,21]. Other reports have shown that aggregated mAb have been found in human biological fluids, including serum [22,23] and plasma [22]. mAb aggregation can, therefore, appear to increase the likelihood of an adverse immune response.

In vitro, cell-based assays that mimic the normal immune response have demonstrated that exposure to aggregated mAbs can induce cell activation, proliferation, and cytokine secretion of immune system components through the activation of dendritic cells [24] and T cells [25,26,27,28]. In vivo, responses to mAb aggregates from animal-based studies showed that heat-aggregated IgG immune complexes lead to cellular activation and immune complex formation [29,30].

In this study, we have used a very sensitive human in vitro skin assay, previously described for predicting adverse immune events to chemicals [31] and small molecule drugs [32], to assess the immunotoxicity and skin sensitization caused by mAb aggregation. This assay is sensitive as it includes dermal exposure of the aggregated monoclonal antibody, T cell activation, cytokine release, and histopathological skin damage as parameters for the detection of adverse immune responses and skin sensitization. In this current study, two commercially available monoclonal antibodies, Herceptin and Rituximab, were subjected to thermal stress conditions to induce aggregation, followed by characterization of protein aggregates. Immune activation was assessed by testing the heat-stressed mAb samples in both T cell proliferation and cytokine release assays alongside a human in vitro skin explant assay [31,32].

## 2. Materials and Methods

### 2.1. Degradation of mAb Samples

Herceptin (Trastuzumab) and Rituximab (Mabthera) monoclonal antibodies (IgG1 class) were used at a concentration of 1 mg/mL and exposed to three different heat stress conditions: 4 °C (fridge), 37 °C (incubator) and 40 °C (water bath) for the following time points: 0, 3, 6, 12, 24 and 48 h. A positive control for aggregation was also prepared for each mAb by leaving the samples at 65 °C for one hour in an acidic buffer (pH3). Samples were given a 3-digit code name for identification, with the first digit representing the mAb tested (Rituximab, R or Herceptin, H), the second digit representing the temperature exposed (4, 37, or 40 °C), and the third digit representing the time of exposure (0, 3, 6, 12, 24 or 48 h). Samples were stored immediately after the heat stress protocol at 4 °C until further analysis.

### 2.2. Protein Analysis of Stressed mAb Samples

The protein content of the heat-stressed mAb samples was quantified by sedimentation velocity-analytic ultra-centrifugation (SV-AUC). Sedimentation analysis was carried out using a ProteomeLab XL-I analytical centrifuge (Beckman Coulter, Palo Alto, CA, USA). The following conditions were used in all centrifugation runs: 40,000-rpm angular velocity, 20 °C rotor temperature, and 280-nm absorbance scanned. Absorbance and interference data were collected for each experiment with a minimum of 65 scans. Protein quantification (percentage of monomers, dimers, and larger molecules) was calculated using SEDNTERP software (2.01). Sedimentation velocity profiles were treated using size-distribution models and refined with Bayesian statistics. Root mean square deviation (RMSD) was also calculated as the sample standard deviation of the differences between predicted and observed values. This quantification was carried out using Newcastle University Protein and Proteome Analysis (NUPPA) service facilities.

Protein characterization was analyzed using transmission electron microscopy (TEM). 10 µL of the mAb stressed samples were deposited on carbon-coated TEM grids, after which the grids were stained with uranyl acetate for negative staining. The grid was air-dried, and the excessive staining was removed from the grid specimens. The grids were then analyzed under an acceleration voltage of 100 kV under a Philips CM100 TEM.

### 2.3. Human Blood and Skin Samples

This study was approved by the Local Research Ethics Committee (LREC). A total of five donor human blood and skin biopsy samples were obtained from healthy volunteers after informed consent by a research nurse at a National Health Service dermatology clinic. Each volunteer donated 60 mL of peripheral blood, collected in heparin (455051, Greiner bio-one), and two 4-mm skin biopsies, which were used for the in vitro skin explant assay. Peripheral blood mononuclear cells (PBMCs) were isolated by density-gradient centrifugation from fresh blood. Skin biopsies (abdominal area) were collected fresh in X-Vivo™ medium (Lonza, Basel, Switzerland) and processed by washing in PBS (Sigma, Burlington, MA, USA), then trimmed of excess fat and dissected into small sections for use in the skin explant assay.

### 2.4. Detection of Aggregated mAb-Induced Immune Activation by T-Cell Proliferation Assay

A 2 × 10^5^ PBMC/well was incubated with aggregated mAb samples at 1 and 10 µg/mL for 3 days. A negative isotype control (IgG1) and a positive control for immune activation (OKT3) [33] were used in each test. Cells were harvested, and [3H] Thymidine was added for the last 16–18 h. [3H] Thymidine primary stock was stored at 37 MBq and used at 3.7 MBq (1/10 dilution), and subsequent uptake was measured using a Micro Beta β-scintillation counter in counts per minute (cpm). Data were interpreted using Prism GraphPad software (V5, Fremont, CA, USA).

### 2.5. Aggregated mAb-Induced Immunotoxicity, including Skin Sensitization Using the Skin Explant Assay

**Figure 1:**
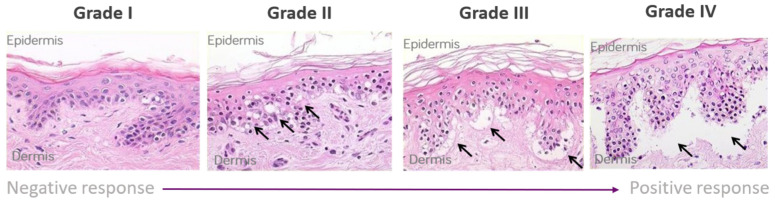
Lerner grading scheme for sub-epidermal lesions in the skin explant assay.

The 4 mm skin biopsies, obtained from volunteers, were cut into multiple smaller sections of approximately 1 mm^2^ for analysis. Each test condition utilized 1 piece of 1 mm^2^ of healthy skin from each volunteer. Skin biopsy sections were incubated in a medium supplemented with autologous, heat-inactivated serum containing both 1 × 10^6^ PBMCs/well and treated mAb samples at a concentration of either 1 or 10 µg/mL for 3 days at 37 °C, 5% CO_2_, 95% humidity. Skin biopsies were then fixed in formalin prior to being paraffin-embedded, sectioned, and stained with haematoxylin and eosin (H&E). Negative controls consisted of co-culture of PBMCs and autologous skin alone and co-culture of the skin with the isotype antibody IgG1. The positive control was OKT3, which has previously been shown to give rise to grade II or III response in skin explant assays [34] Controls were included in all assays. The endpoint of the assay was the assessment of the histopathological damage observed in the skin tissue caused by exposure to the mAb sample. This output was measured as skin grades (grades I to IV) according to the severity of the skin tissue damage observed (Figure 1) [35]. Grade I is considered negative, with an intact upper keratinocyte layer. Grade II includes dyskeratosis and vacuolization of the epidermis and dyskeratotic bodies. Grade III shows more severe damage to the epidermal layer, with the initial separation of the epidermal and dermal layers observed as cleft formation. Grade IV refers to severe damage, with complete separation of the epidermal and dermal layers. Grade II or higher is regarded as a positive response. Grading was performed blind by 2 individual assessors.

### 2.6. Multiplex Cytokine Analysis

Multiplex cytokine analysis was performed on the skin explant assay supernatants using MSD V-Plex kits (Meso Scale Diagnostics, Rockville, MD, USA) following the manufacturer’s instructions. The biomarkers included in this panel were IFN-γ, IL-10, and TNF-α.

### 2.7. Immunofluorescence Labelling

Anti-Hsp70 (Heat-Shock protein 70) (1:20 dilution) antibody (Abcam, Cambridge, UK) was used to detect apoptosis in the skin explant biopsies. Heat shock proteins (HSPs) have a wide array of functions in apoptosis, leading to the suppression of apoptotic pathways. However, stress signals that trigger apoptosis also stimulate the expression and release of HSPs [36]. In this study, we investigated the expression of HsP70, which has been shown in previous studies of skin explants to correlate with increased immune responses [37]. Alexa Fluor 488 (A488, Life Technologies, Carlsbad, CA, USA) was used as the secondary antibody at a concentration of 1 µg/mL. 4′,6-diamidino-2-phenylindole (DAPI) staining was used at a concentration of 1.5 µg/mL, with VECTASHIELD^®^ Mounting Medium (Vector Laboratories, Newark, CA, USA).

### 2.8. Statistical Analysis

Skin explant, T cell proliferation assays, and cytokine assessment were performed using samples from five healthy donors (*n* = 5). The T cell proliferation assay endpoint was a log fold-increase together with Stimulation Indices (SI) in response to exposure to the test compound. SI was measured using the ratio between the test conditions (mAb exposure) and the untreated control (no mAb exposure). The cut-off SI value for a positive response was a log 2-fold increase [32]. Statistical analysis was carried out using repeated measures one-way ANOVA.

For the skin explant test, using the histopathological grading classification, statistical analysis was performed to firstly to compare the mAb concentration (1 and 10 µg/mL) in the same donor by a repeated measures two-way ANOVA with a Bonferroni correction (post-test) and then to see the effect of the different mAb temperature conditions in the same donor by a one-way repeated measures ANOVA with a Bonferroni correction post-test.

Cytokine assessment was analyzed using the MSD Workbench software, then statistically analyzed using a one-way repeated measures ANOVA with a Bonferroni correction post-test.

All statistical tests were carried out using Prism GraphPad software (version 5). Statistical differences were considered significant if *p*-value < 0.05 = *, <0.005 = **.

## 3. Results

In this study, two therapeutic antibodies—Rituximab (chimeric IgG1 mAb) and Herceptin (humanized IgG1 mAb), were exposed to a heat stress protocol. The testing temperatures were intended to mimic storage conditions at 4 °C, 37 °C (normal physiological temperature), and 40 °C (elevated body temperature during an infectious episode). The selected heat stress conditions ensured that mAb degradation mimicked storage or in vivo conditions rather than industrial manufacturing.

### 3.1. Protein Analysis of the Stressed mAb Samples

The aggregated samples were analyzed by SV-AUC to determine the protein content of the heat-stressed mAb samples. Only the control and extreme testing conditions were analyzed at the following time points: 4 °C for 0 h, 37 °C for 48 h, 40 °C for 48 h, and 65 °C for 1 h (positive control for aggregation) (Table 1). Overall, the loss of monomers by heat stress at 4 °C, 37 °C and 40 °C was found to be very low, with up to <6% aggregation of the total protein content, including dimers and larger molecules at 4 °C, 37 °C and 40 °C (Table 1). This result is in concordance with previously reported studies, where a low level (<3%) of total protein content was reported as aggregated [38]. The positive controls (65 °C), however, showed a high loss of monomers of >97% in both instances.

The 4 °C for 0 h was considered as the baseline condition and showed more than 97% of the protein content in the monomer form in both heat-stressed Rituximab and Herceptin. Rituximab showed little variation in the monomer content throughout the different heat stress conditions, with 97.38% monomer content at 37 °C for 48 h and 97.73% at 40 °C for 48 h. Dimer content at baseline was 1.14% of the overall protein content, with a small increase to 1.61% after 37 °C and 1.15% after 40 °C. For the larger molecules, e.g., trimers, tetramers, and heavier molecules, Rituximab demonstrated a small increase from 0.93% at baseline to 1.48% at 37 °C and 1.44% at 40 °C. The decrease in monomer content for Herceptin was more evident. At baseline, the monomer content was 97%, which was reduced to 94.8% at 37 °C and to 95.51% at 40 °C. Correspondingly, the dimer content increased from 1.701% at baseline to 2.854% after 37 °C and 2.611% at 40 °C. There was also an increase in larger molecules, ranging from 0.468% at baseline to 2.653% at 37 °C and to 1.929% at 40 °C.

**Figure 2:**
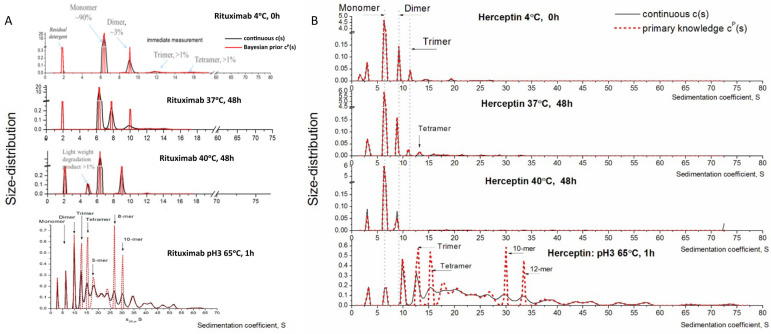
Size distribution based on sedimentation coefficient of the heat-stressed aggregated samples of Rituximab and Herceptin. Quantification of the aggregation state of heat-stressed (**A**) Rituximab and (**B**) Herceptin samples at 4 °C for 0 h, 37 °C for 48 h, 40 °C for 48 h, and 65 °C for 1 h (black line). The results fit the Bayesian prediction model (red dotted line). Monomer species are at around 6S, with elongated dimers appearing at 8S and globular dimers appearing at 10S.

Size distribution of the sedimentation velocity of aggregated Rituximab (Figure 2A) and Herceptin (Figure 2B) showed a good distribution of the monomer, dimer, and larger molecule forms across the different temperature ranges. Size distribution showed that the majority of monomeric species sedimented at 6.3S with a molecular weight of 152 kDa when present in the solution, alongside some dimeric species. The dimeric species had different stoichiometry with both elongated configuration and sedimentation close to 8S and more globular configuration with sedimentation just below 10S. At 37 °C, even larger species were observed with sedimentation up to 15S. This change in protein size reflected the heat stress, causing the mAb to become heavier as it aggregated. As a result, the sedimentation velocity increased when compared to non-aggregated samples.

The internal positive control for aggregation (65 °C incubation for one hour in acidic conditions) aggregated very easily, as expected, with 73% content of larger molecules for Rituximab and 69% for Herceptin. Size-distribution showed a lower number of monomer content and an increased number of trimers, tetramers, and larger molecules (up to 10–15 mers), demonstrating a more aggregated state of this sample.

Overall, the results from the SV-AUC showed that Rituximab was less susceptible to thermodynamic changes when compared to Herceptin, since only Herceptin showed variation in content of monomer, dimer, and large structure molecules throughout the heat stress protocol. These results correlate with the outcome predicted by the Bayesian model.

### 3.2. Visual Characterization of Aggregates

**Figure 3:**
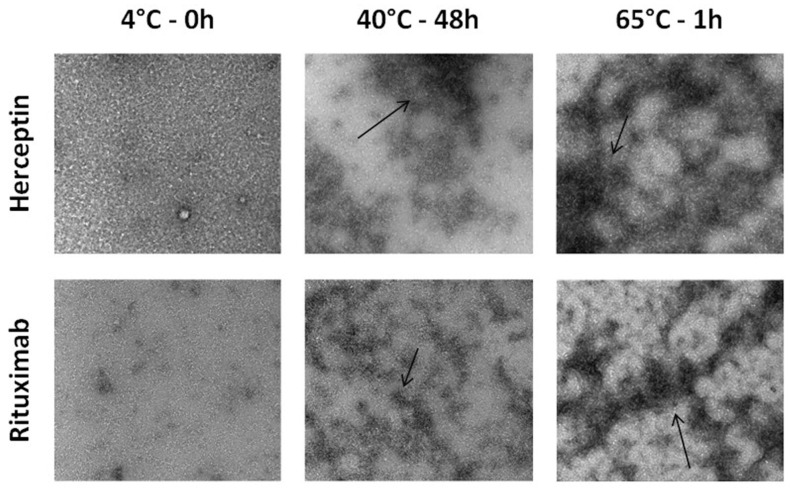
Visual characterization of two heat-stressed mAb samples by Transmission Electron Microscopy (TEM). Comparison of aggregation status of two mAbs, Rituximab and Herceptin, after a heat stress protocol. Increased aggregation highlighted by arrows is shown in heat treated conditions from 4 to 65 °C.

Transmission Electron Microscopy (TEM) was performed to allow visual characterization of the aggregated heat-stressed mAb samples and provide a more qualitative analysis of aggregation. As depicted in Figure 3, the unstressed mAb samples at 4 °C contained almost no microscopic particles, whereas the heat-stressed samples at 40 °C showed small aggregates (black arrows). The number of detectable aggregates was even greater in the positive control for aggregation (65 °C). These results confirm the earlier results reported for protein analysis. Together, the two protein quantification methods indicated that heat stress modified the secondary structure of the mAb and promoted low levels of aggregation. Furthermore, the intensity of the heat stress protocol, as in the case of the positive control at 65 °C, exacerbated the level of aggregation.

When comparing the protein content observed between heat stressed Herceptin and Rituximab, Herceptin showed a slightly increased aggregation level compared to Rituximab. Differences in the primary sequence of these mAbs may explain the differences in the degree of aggregation since Rituximab is a chimeric mAb and Herceptin is a humanized mAb [37].

### 3.3. Aggregated mAb Samples Induce Immune Activation and Skin Sensitization

In view of the observations that heat stress can alter mAb structure and induce aggregation, it was important to understand if this aggregation could lead to immunotoxic responses and potential skin sensitization. The effect of the heat stressed mAbs on immune activation was assessed by a human skin explant assay, T cell proliferation and cytokine release assays. PBMCs and skin biopsies from five healthy donors were stimulated with heat stressed aggregated samples of Rituximab and Herceptin (at 1 and 10 µg/mL) and assessed for increased immune activation.

**Figure 4:**
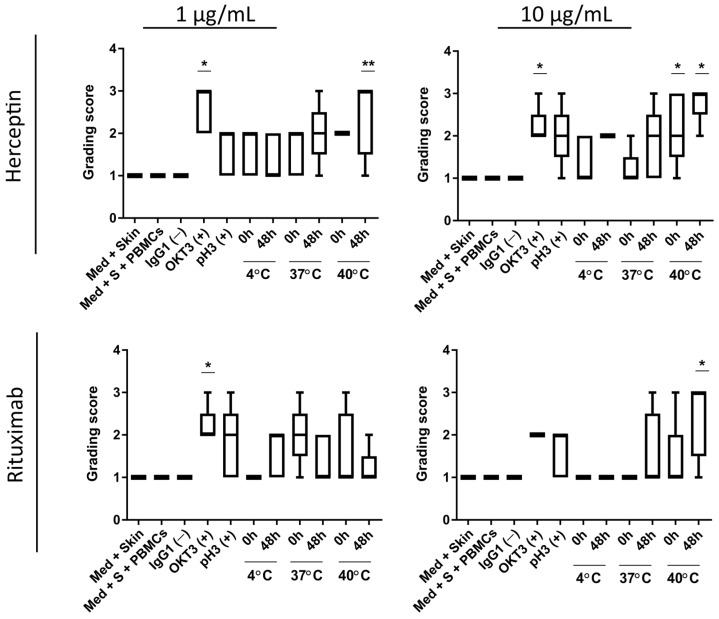
Skin explant assay results from exposure to heat-stressed mAb samples. Heat-stressed samples of Herceptin and Rituximab (1 and 10 µg/mL) were incubated with peripheral blood mononuclear cells (PBMCs) and autologous skin biopsies from healthy donors (*n* = 5) in the skin explant assay. The severity of the histopathological damage was graded using Lerner grades I–IV) Based on the damage observed, the boxes represent the median in the 25th and 75th percentiles with min and max whiskers. A significant increase (** *p* < 0.005 and * *p* < 0.05) in tissue damage response was observed at 1 μg/mL and 10 μg/mL Herceptin when compared to the negative control (skin in medium alone) at the 40 °C/48 h testing condition. Rituximab showed significant tissue damage at 40 °C/48 h when compared to the negative control (* *p* < 0.05). The data were assessed by one-way ANOVA and Bonferroni’s post-hoc test.

**Figure 5:**
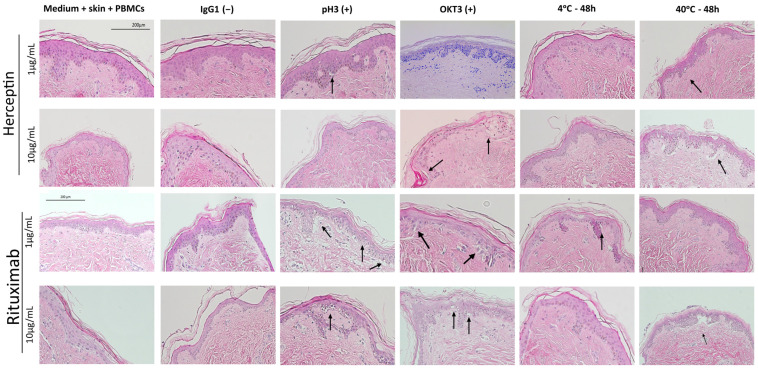
Histopathological damage from exposure to heat-stressed mAb samples. H&E staining of skin tissue after incubation with the heat-stressed samples (60 °C, pH3) of Herceptin and Rituximab at 1 and 10 µg/mL. Black arrows represent histopathological damage on the dermal/epidermal junction in the form of vacuolization of keratinocytes (Grade II positive reaction) and cleft formation leading to separation of the dermis and epidermis (Grade III positive reaction). Scale bars represent 200 µm.

Responses in the skin explant assay showed tissue damage in response to both aggregated Rituximab and Herceptin samples (Figure 4 and Figure 5). Using Lerner’s skin damage classification, skin damage classified as a grade II or higher response is indicative of an adverse immune reaction (Figure 1). At a concentration of 1 µg/mL, Herceptin caused significant tissue damage (*p* < 0.005) with grade III damage in three out of five tests at the 40 °C/48 h testing condition when compared to the negative control (Figure 4).

Herceptin 1 µg/mL tested at 4 °C and 37 °C, caused either a grade I or grade II skin damage response in all tests, with the exception to one test which showed a grade III positive response at 37 °C/48 h. Responses observed at 4 °C and 37 °C were not statistically different from the negative control. Similarly, at 10 µg/mL concentration, Herceptin caused significant tissue damage at 40 °C/48 h (*p* < 0.05), with grade III skin damage observed in four tests and a grade II skin damage response observed in one out of five tests. OKT3, as the positive control for skin damage, caused significant grade III skin damage at both 1 and 10 µg/mL (*p* < 0.05). The positive control for aggregation at 60 °C (pH = 3) caused grade II damage in three out of five tests at both 1 µg/mL and 10 µg/mL and a grade III response in one out of five tests at 10 µg/mL While still considered positive for skin damage, it was, however, not statistically significant from the negative control. These differences in the responses may be related to donor variability.

At 1 µg/mL, Rituximab did not cause any significant skin damage at the testing condition of 4 °C/0 h, where a grade I response was observed in all tests, whereas, at 4 °C/48 h, three of the five tests showed grade II skin damage. At 37 °C/0 h, three tests showed grade II skin damage, and one showed grade III skin damage, whereas, at 37 °C/48 h, only two tests of the five showed grade II skin damage response. At 40 °C/0 h, one test showed a grade II and one test showed a grade III skin damage, whereas, at 40 °C/48 h, only one test showed a positive response (grade II). OKT3 caused significant damage (*p* < 0.05), with a grade II response in four out of five tests and a grade III response in one test. At 10 µg/mL, Rituximab tested at 4 °C/0 h, 4 °C/48 h, and 37 °C/0 h showed a negative (grade I) response in all five tests. At 37 °C/48 h, a grade III response was observed in one test, and a grade II response was observed in one test of the five tests. At 40 °C/0 h, one test showed a grade III response, whereas the remaining four of the five tests showed a negative response. At 40 °C/48 h, a grade III response was observed in three tests, and a grade II response was observed in 1 out of five tests. Grade II positive responses were observed in response to OKT3.

The fact that Herceptin caused significant skin damage at both 1 and 10 µg/mL while Rituximab only caused significant damage at 10 µg/mL, suggested aggregated Herceptin and Rituximab have different immunotoxic potency profiles due to their aggregation status.

**Figure 6:**
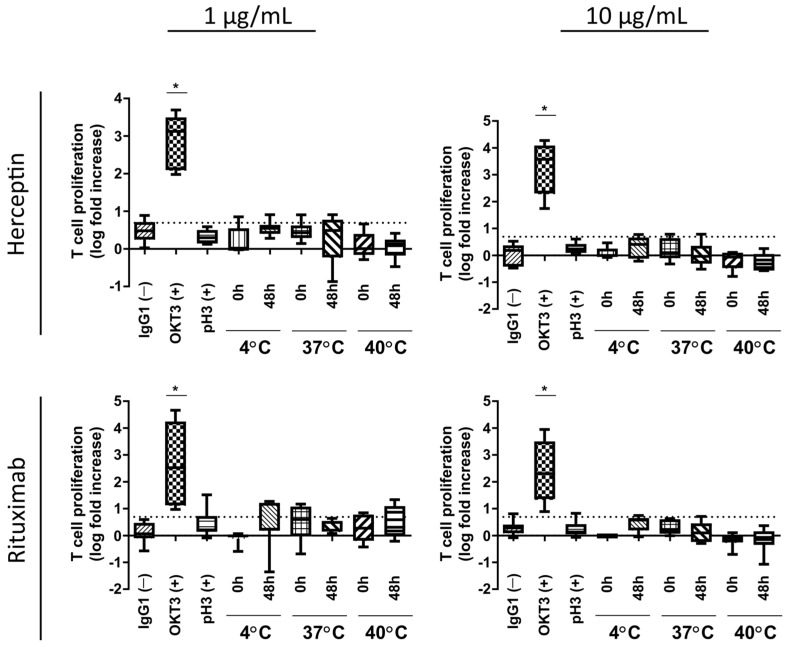
T-cell proliferation assessment resulting from exposure to heat-stressed mAb samples. Heat-stressed samples of Herceptin and Rituximab (1 and 10 µg/mL) were incubated with donor-derived PBMC (*n* = 5 for each condition). T-cell proliferation assessment alone was not capable of detecting an increased immunological profile induced through protein aggregation. The boxes represent the median with the 25th and 75th percentile with min and max whiskers. * *p* < 0.05 Bonferroni’s post-hoc test after One-way ANOVA. The log 2-fold positive threshold is indicated by the dotted line.

Figure 5 shows representative H&E images of each of the treatment conditions for Rituximab and Herceptin. Black arrows indicate histological changes on the dermal/epidermal junction relevant to the grading process, namely epidermal disruption. T cell proliferation assays showed no significant increase in T cell proliferation upon exposure to aggregated Herceptin or Rituximab (Figure 6) in any of the testing conditions. The positive control, OKT3, elicited a T cell stimulation index above the log 2-fold threshold in both mAbs at 1 and 10 µg/mL.

**Figure 7:**
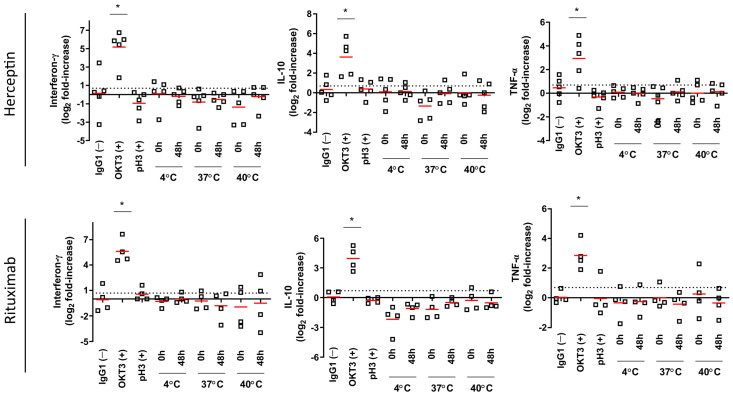
Multiplex cytokine panel analysis of experimental conditions tested at 1 µg/mL. For each condition (*n* = 6), the median is represented by a red line. The log 2-fold positive threshold is indicated by the dotted line. * *p* < 0.05 Bonferroni’s post-hoc test after One-way ANOVA.

**Figure 8:**
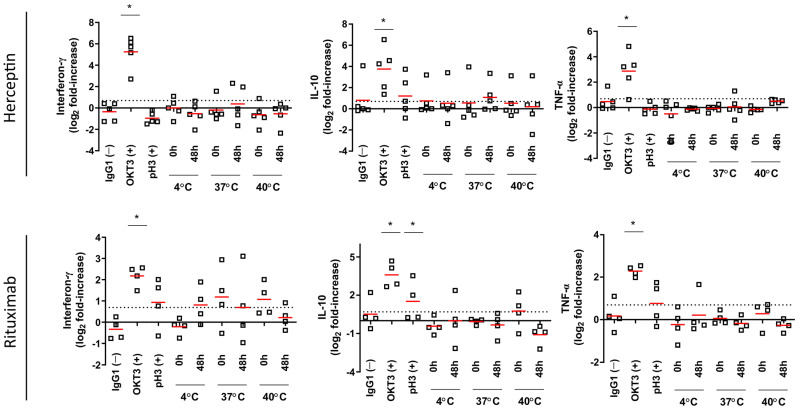
Multiplex cytokine panel analysis of experimental conditions tested at 10 µg/mL. For each condition (*n* = 6), the median is represented by a red line. The log 2-fold positive threshold is indicated by the dotted line. * *p* < 0.05 Bonferroni’s post-hoc test after One-way ANOVA.

Cytokine levels measured in cell culture supernatants from samples tested with 1 µg/mL (Figure 7) and 10 µg/mL (Figure 8) showed that IL-10, IFN-γ, and TNF-α levels were significantly elevated after exposure to OKT3 at both test concentrations (*p* < 0.05). Aggregated Rituximab and Herceptin tests, when compared to IgG1 controls, showed no significant increase in cytokine levels at both 1 and 10 µg/mL. However, increased yet non-significant IFN-γ levels were observed in response to 10 µg/mL in the Rituximab test when compared to the negative control. The positive control for aggregation (pH3) showed non significantly increased IL-10 levels when tested at 10 µg/mL (Figure 8) in response to Herceptin compared to a significant increased in response to Rituximab (*p* < 0.05).

**Figure 9:**
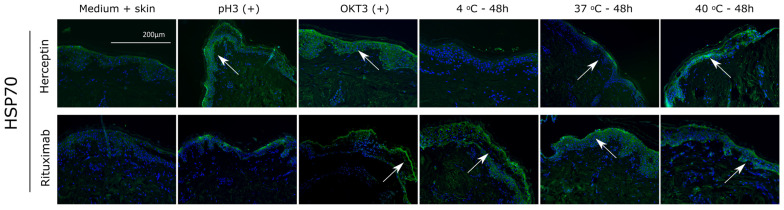
Apoptosis caused by mAb aggregation. Heat stressed aggregated samples of Herceptin and Rituximab were stained for heat shock protein 70 (HSP70) by immunofluorescence. DAPI was used as nuclear marker (blue). White arrows indicate areas of positive staining.

While no significant increases in T cell proliferation responses were observed upon exposure to heat-stressed mAbs, histopathological damage showing grade II and III positive responses were observed in the in vitro skin explant assay. To determine if apoptosis was occurring within the tissue sections, Heat Shock Protein (HSP) 70 staining was performed (Figure 9). Positive HSP 70 staining (bright green) was observed in the 37 °C and 40 °C conditions in both Herceptin and Rituximab at 10 µg/mL. Positive control for aggregation at 60 °C (pH3) also showed positive expression of HSP70.

## 4. Discussion

Previous studies addressing the immunotoxic potential of mAb aggregation have used heat stress protocols with temperatures as high as 65 °C (25), 70 °C (26), or 80 °C [24,33]. In comparison, the maximum temperature condition set for this study was 40 °C. The temperature test points were chosen for their relevance to an in vivo scenario based on normal and fever-induced body temperatures. Our results demonstrated that temperature treatment of both Herceptin and Rituximab induced changes in the aggregative status of the tested samples. It could be suggested that a level of protein unfolding and consequent rearrangement of the mAb secondary structure was primarily responsible for the presence of larger molecules in the aggregated samples (as demonstrated by SV-AUC results). TEM analysis of aggregated mAbs demonstrated this effect visually, with clear changes in aggregation observed in heat-treated samples, especially at higher temperatures of 65 °C. These thermodynamic changes accounted for less than 4% aggregation of the total protein content, which is in accordance with previous reports of 3% aggregation [26] of the total protein content under heat stress conditions.

There is evidence that protein aggregation can occur at different stages of mAb production or during later storage, imposing a health risk to patients upon administration of therapeutic mAbs [38]. We have studied the potential immunotoxic effect of mAb aggregation by investigating histopathological assessment of cell damage in a novel in vitro skin explant assay, T cell proliferation, and cytokine release. Overall, our data shows heat stressed Herceptin and Rituximab samples did not enhance T cell proliferation rates. T cell proliferative induction is primarily stimulated through increased dendritic cell activity [28]. Our assay could be improved through the incorporation of dendritic cell activity alongside T cell proliferative responses. This would allow us to gain a more complete assessment of the ongoing processes within the assay and provide an additional data point to further improve the validity of our predictive outcome. Furthermore, the composition of PBMC populations can vary significantly across individuals. In this study, PBMC populations were not assessed and normalized prior to use in stimulatory experiments. Characterization of the PBMC population prior to use within the assay could allow for a level of data normalizing, which could further improve assay reliance and sensitivity.

A trend for elevated expression of IFN-γ was observed at 10 µg/mL in Rituximab test conditions. IL-10 is an immunosuppressive cytokine which inhibits expression of pro-inflammatory cytokines [38]. Elevated expression of IL-10 in the 10 µg/mL Rituximab positive control could suggest that cells are actively forming a protection mechanism from the damage caused by the aggregated mAbs, by dampening the immune response and sustaining normal tissue homeostasis.

Previously published data [31,32] based on the human tissue explant assay used in this study has resulted in convincing prediction of adverse immune events in response to chemical [31] and small molecule drug stimulation [32]. The histopathological damage assessment shown here suggests that heat-stressed Rituximab and Herceptin do cause some degree of immune activation. These results do not fully corroborate previously published studies, where it was shown that mAb aggregation by heat stress elicits in vitro innate and late-stage T cell responses [27], activates dendritic cells to stimulate T cells [24] and induces CD4+ T cell proliferation via pro-inflammatory cytokine release [25].

Heat shock protein 70 (HSP70) serves as a molecular chaperone in cases of cell stress (as heat shock or oxidative stress). HSP70 is crucial for cell survival after apoptotic stimuli [39,40], as shown by Mosser et al. [40] in which macrophages heat-stressed for up to two hours at 43 °C, showed protection of DNA fragmentation in the presence of HSP70 [39]. HSP70 was shown to be significantly upregulated in all treated samples and absent in negative controls. This suggests that some level of inflammatory stress was being imparted throughout the assay conditions. HSP70 is most likely involved in the protein refolding after exposure to the aggregated Herceptin and Rituximab, and its overexpression may rescue cells from apoptotic cell death. Skin explant assay samples were tested at 4 °C, 37 °C and 40 °C temperature conditions. Centrifugal analysis of aggregate formation at these temperatures only showed relatively moderate changes in aggregated antibody levels with the vast majority of mAbs still present in the monomer formation. This suggests that, at best, only a mild immune stimulatory effect would be observed and that this, alongside HSP70’s anti-apoptotic effects, may partly explain why mild histological damage was observed in aggregated antibody-treated conditions.

Overall, only the highest testing temperature (40 °C) caused significant histopathological damage in comparison to the baseline condition whereby grade III skin damage was observed in most of the donors, while at 4 °C and 37 °C an overall grade II was observed. While the outcome is not a severe grade, these results still show positive skin damage caused by heat-stressed aggregated mAbs. The higher condition of heat stress (40 °C) did cause more severe histopathological damage than the positive control (pH3). We could hypothesize that highly aggregated mAbs (as in the case of the positive control for aggregation, pH = 3) can affect the normal binding of these antibodies to the antigen-presenting cells [24]. It has been shown that the structural mAb rearrangements that result from exposure to temperature shock can lead to neo-epitopes [41] (new epitopes formed during aggregation), glycosylation/pegylation changes [42] and other post-translational modifications that break tolerance towards these mAbs, thus causing immunotoxicity. Furthermore, it has been shown that only covalently modified aggregates can induce immunotoxicity [29]. However, for aggregated mAbs to cause an immunotoxic response, it is vital that conformation of the protein’s active site remains preserved regardless of aggregation [43].

Thus, we can speculate that the low pH (pH3) internal positive control denatured the antibodies to such an extent that protein conformation was lost by denaturation during heat stress. The consequential aggregation occurring after denaturation impeded the preservation of the protein’s active site. These results also highlight the fact that protein aggregation does not always directly imply immunotoxicity. While structural changes might occur due to aggregation, it does not mean that protein functionality is compromised. Previous studies showed that immunotoxicity is only achieved if the primary structure of the mAb protein is deeply modified after aggregation stress [23]. Therefore, it can be argued that the positive low pH control for aggregation did not cause a high level of immune activation/cell death due to the new structural rearrangement of the protein, hiding its active site.

Further work is required to better understand the potential use of the skin explant assay in the assessment of the immunotoxicity of aggregated antibodies. The data presented here represents a small study looking at relatively few aggregative conditions and only 2 mAbs. Nevertheless, it should still be emphasized that this novel assay was able to detect some immune activation through histological damage assessment directly brought about by immune cell activity within the assays. Further work is required to elucidate the mechanistic actions around aggregate formation and the effect of aggregate presence on immune cell activation. Future work involving this assay should focus on expanding the donor replicates within the explant assays and should include a greater range of temperatures to induce aggregate formation. Additionally, a larger sample size of mAbs based on different therapeutic areas and mechanistic action could elucidate additional areas of study interest.

## 5. Conclusions

This study was aimed at testing a novel in vitro test method for assessment of skin sensitization and adverse immune events relevant to human safety and has provided new insights for potentially reducing animal testing in the future. Our method was able to predict immune activation facilitated through temperature-mediated mAb aggregate formation in two antibody systems with a reasonable level of accuracy.

## 6. Patents

Alcyomics holds patents on the skin explant assay (trading as Skimune®) see table
**Case Code****Applicant****Country****Status****Official Number****Title****P131663CH**Alcyomics LimitedSwitzerlandGranted‘EP2524227Skin Explant Assay**P131663DE**Alcyomics LimitedGermanyGranted‘602011004834Skin Explant Assay**P131663DK**Alcyomics LimitedDenmarkGranted‘EP2524227Skin Explant Assay**P131663ES**Alcyomics LimitedSpainGranted‘EP2524227Skin Explant Assay**P131663FR**Alcyomics LimitedFranceGranted‘EP2524227Skin Explant Assay**P131663GB1**Alcyomics LimitedUnited KingdomGranted‘EP2524227Skin Explant Assay**P131663IT**Alcyomics LimitedItalyGranted‘502014902254005Skin Explant Assay**P131663US**Alcyomics LimitedUSAGranted‘9,651,544Skin Explant Assay**P131663USD1**Alcyomics LimitedUSAGranted‘10,073,084Skin Explant Assay

## Figures and Tables

**Table 1:** Quantification of the aggregated content of heat-stressed mAb samples by analytical ultra-centrifugation (SV-AUC).

			Monomer			Dimer			Larger Molecules	RMSD
	Temp (°C)	Time (h)	Sedimentation (S)	Mass (kDa)	%	Sedimentation (S)	Mass (kDa)	%	%	
Rituximab	4	0	6.412	152.1	97.92	9.25	263.653	1.14	0.93	0.015
		48	6.415	138.3	97.72	9.715	257.707	1.7	0.58	0.017
	37	0	6.411	153.4	96.16	9.089	258.781	2.69	1.15	0.014
		3	6.411	149.9	97.41	9.27	274.93	1.68	0.91	0.013
		6	6.408	152.5	96.88	9.19	261.938	2.07	1.05	0.011
		12	6.413	149.3	96.69	9.522	270.064	2.28	1.11	0.010
		24	6.426	146.8	99	-	-	-	1	0.018
		48	6.424	148.3	97.38	8.34	229.272	1.61	1.48	0.014
	40	0	6.415	153	97.26	9.042	262.435	2.23	1.24	0.016
		3	6.407	147.4	98.34	8.997	262.004	1.18	1.3	0.017
		6	6.399	149.5	98.16	8.768	249.582	1.15	1.04	0.017
		12	6.397	155.3	98.40	-	-	-	1.60	0.017
		24	6.425	150.3	97.38	9.461	281.061	1.32	1.42	0.018
		48	6.42	147.6	97.73	9.649	281.75	1.15	1.44	0.016
	65	1	1.587	13.11	0.706	6.503	108.75	2.544	73.01	-
Herceptin	4	0 *	6.441	149	97.96	9.432	263.977	1.701	0.468	0.010
	37	48	6.427	148.1	94.8	9.109	249.839	2.854	2.653	0.010
	40	48	6.421	148.1	95.51	9.235	255.364	2.611	1.929	0.011
	65	1	6.346	132.6	2.661	9.862	256.928	6.107	69.246	0.010

Quantification of monomers, dimers, and larger molecules in Rituximab and Herceptin monoclonal antibodies (at 1 mg/mL) after exposure to a heat-stress protocol (4, 37, and 40 °C for 0, 3, 6, 12, 24, and 48 h). Time 0 * for Herceptin was identical for the temperature ranges (4–40 °C). Quantification by sedimentation velocity (S), mass (kDa), and percentage in overall mAb sample (%) based on absorbance data. Standard deviation is calculated as Root Mean Square Deviation (RMSD). Missing values (-).

## Data Availability

The original data presented in the study are included in the article; further inquiries can be directed to the corresponding author.
